# Genetic polymorphisms of cytochrome P450 19 and 1B1, alcohol use, and breast cancer risk in Korean women

**DOI:** 10.1038/sj.bjc.6600761

**Published:** 2003-03-04

**Authors:** K-M Lee, J Abel, Y Ko, V Harth, W-Y Park, J-S Seo, K-Y Yoo, J-Y Choi, A Shin, S-H Ahn, D-Y Noh, A Hirvonen, D Kang

**Affiliations:** 1 Department of Preventive Medicine, Seoul National University College of Medicine, Cancer Research Institute, 28 Yongon-Dong Chongno-Gu, Seoul 110-799, Korea; 2 Department of Experimental Toxicology, Research Institute of Environmental Health, Heinrich-Heine-University, Auf'm Hennekamp 50, 40225 Düsseldorf, Germany; 3 Department of Internal Medicine, University of Bonn, Wilhelmstreet 35-37, 53111 Bonn, Germany; 4 Department of Biochemistry, Seoul National University College of Medicine, 28 Yongon-Dong Chongno-Gu, Seoul 110-799, Korea; 5 Department of Surgery, Ulsan University College of Medicine, 388-1 Pungnab-Dong Songpa-Gu, Seoul, Korea; 6 Department of Surgery, Seoul National University College of Medicine, 28 Yongon-Dong Chongno-Gu, Seoul 110-799, Korea; 7 Finnish Institute of Occupational Health, FIN-00250 Helsinki, Finland

**Keywords:** *CYP19*, *CYP1B1*, alcohol, breast cancer

## Abstract

A case–control study was performed to assess the potential influence of *CYP19* Arg^264^Cys and *CYP1B1* Leu^432^Val polymorphisms on breast cancer risk in a series of Korean breast cancer patients and controls. The results suggest that the *CYP19* Arg^264^Cys polymorphism modifies breast cancer risk (OR=1.5, 95% CI=1.1–2.2), especially in association with alcohol consumption (*P* for interaction=0.04), whereas the *CYP1B1* Leu^432^Val polymorphism appears to play no role here.

Breast cancer is the second most frequent cancer in Korean women and its incidence is increasing (Yoo *et al*, 1998). Lifetime cumulative exposure to oestrogens is known as the most important risk factor for breast cancer (Yager, 2000). A variety of different enzymes are involved in the synthesis of oestrogen from cholesterol and further metabolism of oestrogen (Thompsen and Ambrosone, 2000). Polymorphisms of genes encoding for these proteins are regarded as the candidates for elevated breast cancer risk.

The *CYP19* gene encodes aromatase, which catalyses the formation of oestrogens from testosterone and androstenedione. To date, several polymorphisms have been found in the *CYP19* gene (i.e. Polymeropulous *et al*, 1991; Sourdaine *et al*, 1994; Siegelmann-Danieli and Buetow, 1999; Healey *et al*, 2000; Miyoshi *et al*, 2000). One of these, a C-to-T variation in exon 7 resulting in an Arg^264^Cys amino-acid exchange, has been shown to be very common in Asians (Watanabe *et al*, 1997; Miyoshi *et al*, 2000) and could thus be an important modifier of breast cancer risk in this ethnic group.

The CYP1B1 enzyme is known to be involved in the formation of 4-hydroxyoestradiol, which is a catechol metabolite of oestrogen (Hayes *et al*, 1996). A C-to-G variation in exon 3 of the *CYP1B1* gene results in a Leu^432^Val amino-acid exchange. The *Leu/Leu* genotype has been associated with increased breast cancer risk in an Asian population (Zheng *et al*, 2000) but controversial results have been reported as well (Bailey *et al*, 1998; Watanabe *et al*, 2000).

In this study, we have evaluated the potential influence of *CYP19* Arg^264^Cys and *CYP1B1* Leu^432^Val polymorphisms on the breast cancer risk among Korean women as an extension of our previous work in this study population (Park *et al*, 2000; Yim *et al*, 2001).

## MATERIALS AND METHODS

### Study subjects

The criteria of subject selection and details of data collection on lifestyle have been described elsewhere (Park *et al*, 2000; Yim *et al*, 2001). Eligible study population consisted of 389 incident breast cancer cases and 346 controls with no other known cancer or systemic disease, admitted in 1995–2001 to three teaching hospitals located in Seoul, Korea (Seoul National University Hospital, Borame Hospital, and Asan Medical Center). Each patient was frequency-matched to one control in the following age groups: under 30, 30–34, 35–39, 40–44, 45–49, 50–54, 55–59, 60–69, and over 70 years; 288 cases and 288 controls were selected and genotyped for *CYP19*. Informed consents were obtained at the time of blood withdrawal. Information on demographic characteristics, education, marital status, reproductive factors and menstruation, family history of breast cancer in the first and second relatives, lifestyle habits (including smoking, alcohol drinking, and diet) was collected using a questionnaire administered by trained interviewers.

### Genotyping

DNA was isolated using standard methods from blood drawn into 10 ml heparinised tubes and stored at −70°C until use. The *CYP19* genotypes were determined by dynamic allele-specific hybridisation system (DASH, Hybaid). Briefly, a 63 bp long amplification product was obtained using about 10 ng DNA as template in a polymerase chain reaction (PCR) with 20 pmol of oligonucleotide primer P1 (5′-TGC CAT AGA AGT TCT GAT AGC-3′) and 4 pmol of primer P2 (biotin-labelled 5′-TCT TCC AGT TTC TCT TCT GT-3′) (Bioneer: Seoul, Korea) in a total volume of 20 *μ*l. The amplification conditions were: initial denaturation at 95°C for 5 min followed by 45 cycles of 15 s at 94°C and 30 s at 58°C (Multiblock System, Hybaid). The PCR products were hybridised with the following probes: 5′-AAA AAA GA**C** GCA GGA TT-3′ and 5′-AAA AAA GA**T** GCA GGA TT-3′. The *CYP1B1* was genotyped for 241 cases and 290 controls recruited from 1995 to 1999 by a real-time PCR-based method as described by Brüning *et al* (1999).

### Statistical analyses

Odds ratios (ORs) and 95% confidence intervals (CIs) were estimated by unconditional logistic regression analysis adjusting for age, education, body-mass index, age at first full-term pregnancy, family history of breast cancer, duration of breast feeding, and alcohol consumption. Information on alcohol consumption was collected by three questions: (1) How frequently do you drink alcohol (i.e., per week, month, year)? (2) How long have you been drinking? (3) Have you ever quitted drinking? Alcohol intake was categorised as: never-drinking (never and less than once per month) or ever drinking (at least once per month). Interactions between known risk factors (alcohol consumption and age at first full-term pregnancy) and each genotype were evaluated by likelihood ratio test. The difference of two −2log L values of logistic models with and without interaction terms was referred to tables of *χ*^2^ on one degree of freedom.

## RESULTS

The distribution of established breast cancer risk factors was compared in cases and controls (Table 1

**Table 1:** Selected characteristics for matched breast cancer cases and control subjects

	**Cases**	**Controls**	**OR**
	***N* (%)**	***N* (%)**	**(95% CI)^a^**
*Age*			
⩽39	79 (27.4)	79 (27.4)	
40–49	90 (31.3)	90 (31.3)	
50–59	74 (25.7)	74 (25.7)	
⩾60	45 (15.6)	45 (15.6)	
			
*Education*			
Under high school	154 (54.2)	204 (71.6)	1.0
At and over high school	130 (45.8)	81 (28.4)	2.1 (1.5–3.0)
			
*Age at first full-term pregnancy (FFTP)*			
FFTP<30	242 (84.0)	259 (90.2)	1.0
Nulliparous or FFTP⩾30	46 (16.0)	28 (9.8)	1.8 (1.1–2.9)
			
*Family history of breast cancer in first and second degree relatives*			
No	263 (91.3)	278 (96.5)	1.0
Yes	25 (8.7)	10 (3.5)	2.6 (1.2–5.6)
			
*Alcohol consumption*			
<1/month	208 (72.2)	229 (79.5)	1.0
⩾1/month	80 (27.8)	59 (20.5)	1.5 (1.0–2.2)
			
*Cigarette smoking*			
<400 cigarettes/lifetime	270 (93.8)	271 (94.1)	1.0
⩾400 cigarettes/lifetime	18 (6.3)	17 (5.9)	1.1 (0.5–2.1)

a ORs were not adjusted for other covariates.

). These two groups differed statistically significantly in education (OR=2.1, 95% CI=1.5–3.0), age at first full-term pregnancy (OR=1.8, 95% CI=1.1–2.9), family history of breast cancer in first and second degree relatives (OR=2.6, 95% CI=1.2–5.6), and alcohol consumption (OR=1.5, 95% CI=1.0–2.2). The distributions of *CYP19* and *CYP1B1* genotypes are shown in Table 2

**Table 2:** The distributions of *CYP19* Arg^264^Cys and *CYP1B1* Leu^432^Val genotypes and the respective breast cancer risks

	**Cases**	**Controls**	**OR**
	***N* (%)**	***N* (%)**	**(95% CI)^a^**
*CYP19*			
Arg/Arg	150 (52.1)	176 (61.1)	1.0
Arg/Cys	134 (46.5)	106 (36.8)	1.5 (1.1–2.2)
Cys/Cys	4 (1.4)	6 (2.1)	1.0 (0.3–3.9)
Arg/Arg	150 (52.1)	176 (61.1)	1.0
Arg/Cys or Cys/Cys	138 (47.9)	112 (38.9)	1.5 (1.1–2.2)
			
*CYP1B1*			
Leu/Leu	188 (78.0)	229 (79.0)	1.0
Leu/Val	47 (19.5)	56 (19.3)	1.0 (0.7–1.6)
Val/Val	6 (2.5)	5 (1.7)	1.1 (0.3–4.7)
Leu/Leu	188 (78.0)	229 (79.0)	1.0
Leu/Val or Val/Val	53 (22.0)	61 (21.0)	1.0 (0.7–1.6)

a ORs adjusted for age, education, alcohol consumption, body-mass index, family history of breast cancer, age at first full-term pregnancy, and duration of breast feeding.

. The genotype distributions in the control subjects agreed with those predicted by the Hardy–Weinberg equilibrium. The frequency of *CYP19 Cys* allele-containing genotypes (39%) was somewhat lower than that previously found in Japanese women (52–54%) (Watanabe *et al*, 1997; Miyoshi *et al*, 2000), whereas the frequency of *CYP19* *Cys* allele-containing genotypes was very low, less than 10% for Caucasians (Siegelmann-Danieli and Buetow, 1999; Healey *et al*, 2000). The frequency of *CYP1B1* *Val* allele-containing genotype (21%), on the other hand, was similar to that (29%) found in Japanese women (Watanabe *et al*, 2000). However, it was rather surprisingly lower than that found in Chinese women (85%) (Zheng *et al*, 2000), Caucasians (70–81%), and African Americans (95%) (Bailey *et al*, 1998; Ko *et al*, 2001).

The *CYP19* *Cys* allele-containing genotypes showed a significantly increased risk of breast cancer (OR=1.5, 95% CI=1.1–2.2), whereas the *CYP1B1* *Val* allele-containing genotypes had no effect in this context (OR=1.0, 95% CI=0.7–1.6) (Table 2).

When the genotype effects were evaluated in relation with the known risk factors for breast cancer (alcohol consumption, age at first full-term pregnancy), a significant interactive effect was observed between *CYP19* genotype and alcohol consumption (*P* for interaction=0.044). Ever-drinking women with *CYP19* *Cys* allele-containing genotypes showed a 3.3-fold risk (95% CI=1.7–6.5) for breast cancer compared with never-drinking women with the *Arg/Arg* genotype (Table 3

**Table 3:** The ORs and 95% CIs for *CYP19* genotypes in relation to alcohol consumption^a^

	**Arg/Arg**	**Arg/Cys or Cys/Cys**
	**(cases/controls)**	**(cases/controls)**
Alcohol consumption		
<1/month	1.0	1.2 (0.8–1.9)
	(111/134)	(97/95)
⩾1/month	1.1 (0.7–1.9)	3.3 (1.7–6.5)^b^
	(39/42)	(41/17)

a ORs adjusted for age, education, body-mass index, family history of breast cancer, age at first full-term pregnancy, and duration of breast feeding:

b *P* for interaction=0.044.

).

In contrast to the *CYP19* genotypes, no interaction was observed between the known risk factors for breast cancer and the *CYP1B1* genotypes. Neither was there a gene–gene interaction between the *CYP19* and *CYP1B1* genotypes.

## DISCUSSION

Our results suggest that both *CYP19* genotype and alcohol consumption play important roles in breast cancer development, and that these factors could synergistically increase the risk of breast cancer in Korean women. The contrasting findings with previous studies (Watanabe *et al*, 1997; Siegelmann-Danieli and Buetow, 1999; Healey *et al*, 2000; Miyoshi *et al*, 2000) may be because of the differences in subject selection (noncomparable controls in the above studies), sample size (less than 200 in both cases and controls), and inadequate statistical power, marked differences in frequency of *Cys* allele (52–54% for Japanese women, 39% for Korean women, and less than 10% for Caucasian), and different genotyping methods (single-strand conformation polymorphism, sequencing, and dynamic allele-specific hybridisation).

The Arg^264^Cys polymorphism is located in or near recognition site of CYP19 aromatase and thus, it might enhance the oestrogen synthesis and exposure to endogenous oestrogen. However, it is also be possible that *CYP19* Arg^264^Cys polymorphism is in linkage disequilibrium with other important polymorphic site such as TTTA repeat polymorphism (Kristensen *et al*, 1998; Haiman *et al*, 2000; Baxter *et al*, 2001). Watanabe *et al* (1997) reported, however, that Arg^264^Cys genotype did not affect the aromatase activity *in vitro* test so further studies on the potential role of this polymorphism in breast cancer aetiology are required.

One mechanism by which alcohol consumption may increase breast cancer risk is through increased circulating oestrogen and androgen levels (Singletary and Gapstur, 2001). In a controlled feeding study, serum levels of estrone sulphate and dehydroepiandrosterone sulphate were significantly increased by the consumption of 30 g of alcohol per day in postmenopausal women (Dorgan *et al*, 2001). Since *CYP19* is also involved in the biosynthesis of oestrogen, the *CYP19* genotype and alcohol consumption may synergistically increase breast cancer risk by affecting both synthesis and metabolism of oestrogen.

In contrast to the *CYP19* genotype, we found no association between *CYP1B1* genotype and breast cancer. While our finding is consistent with some previous studies (Bailey *et al*, 1998; Watanabe *et al*, 2000), Zheng *et al* (2000) reported that Chinese women with *Leu/Leu* genotype had a 2.3-fold elevated risk of breast cancer among 186 cases and 200 controls. This inconsistency might at least partly be explained by the remarkable difference in the frequency of *Val* allele-containing genotypes (21 *vs* 85%).

In conclusion, the results of this study support the hypothesis that *CYP19* genotype and alcohol consumption play important roles in breast cancer development and that these factors may synergistically increase the risk of this malignancy in Korean women. Epidemiological studies with large sample sizes are, however, required to confirm these preliminary findings and to evaluate the role of the other *CYP19* genotypes of potential interest in breast cancer development in this ethnic group.
